# A Novel Unidirectional Porous β-tricalcium Phosphate Bone Substitute in Orthopedic Surgery: A Technical Note and Case Illustrations

**DOI:** 10.7759/cureus.7465

**Published:** 2020-03-30

**Authors:** Toru Funayama, Hiroshi Noguchi, Hiroshi Kumagai, Tomokazu Yoshioka, Masashi Yamazaki

**Affiliations:** 1 Orthopaedic Surgery, University of Tsukuba, Tsukuba, JPN

**Keywords:** β-tricalcium phosphate, orthopedic surgery, bone graft, artificial bone, unidirectional porous structure

## Abstract

Bone grafting is frequently performed in orthopedic surgeries. Artificial bone is a common grafting material. We have developed an absorbable material for bone regeneration with a unique structure: unidirectional porous β-tricalcium phosphate (Affinos^Ⓡ^;^ ^Kuraray Co., Ltd., Tokyo, Japan). The most distinctive feature of this material is the ease with which blood can rapidly reach its depths by capillary action due to the unidirectional porous structure. It is also characterized by the presence of micropores, which are known to be beneficial for osteoconductivity both on the surface and inside the material. Favorable artificial bone absorption and regeneration with natural bone were observed in cases of clinical bone graft applications in the spine, extremities, benign bone tumor, trauma, and donor sites. Affinos is useful as a novel absorbable material for bone regeneration in various orthopedic surgeries.

## Introduction

Bone grafting is frequently used in orthopedic surgery [1]. The commonly used types of bone grafts vary from country to country. In Japan, these are autologous (56.4%), artificial (40%), and allogenic bone grafts (3.6%) [2]. Allogenic bone grafting is used in some cases, mainly for the supplementation of large bone defects, such as in revision total hip arthroplasty in Japan, and the trend remains unchanged these days [3]. Only a limited amount and quality of autologous bone can be harvested from osteoporotic patients, whose numbers have been increasing due to the rapid aging of the population in recent years. Therefore, artificial bones are largely used to meet the needs of bone grafting during orthopedic surgery.

## Technical report

**Figure 1:**
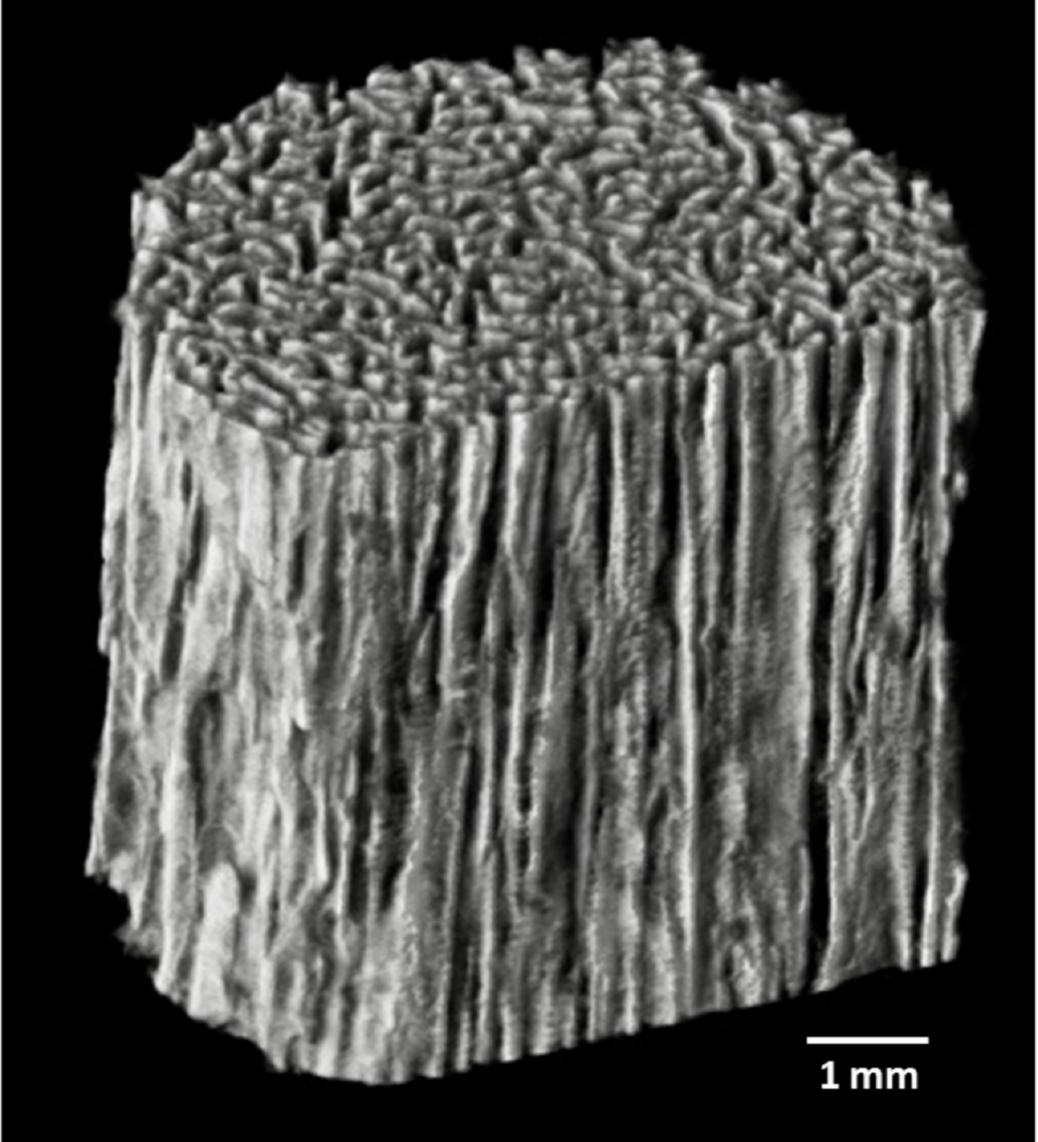
3D-CT image of Affinos Affinos has a unique, characteristic structure in which unidirectional pores 25-300 μm in diameter are aligned in one direction. Affinos: Kuraray Co., Ltd., Tokyo, Japan

We have developed a unidirectional porous β-tricalcium phosphate (β-TCP) material, Affinos^Ⓡ^ (Kuraray Co., Ltd., Tokyo, Japan) for use as an artificial bone with a unidirectional porous structure in which efficient tissue penetration and high osteoconductivity can be expected (Figure 1).

**Figure 2:**
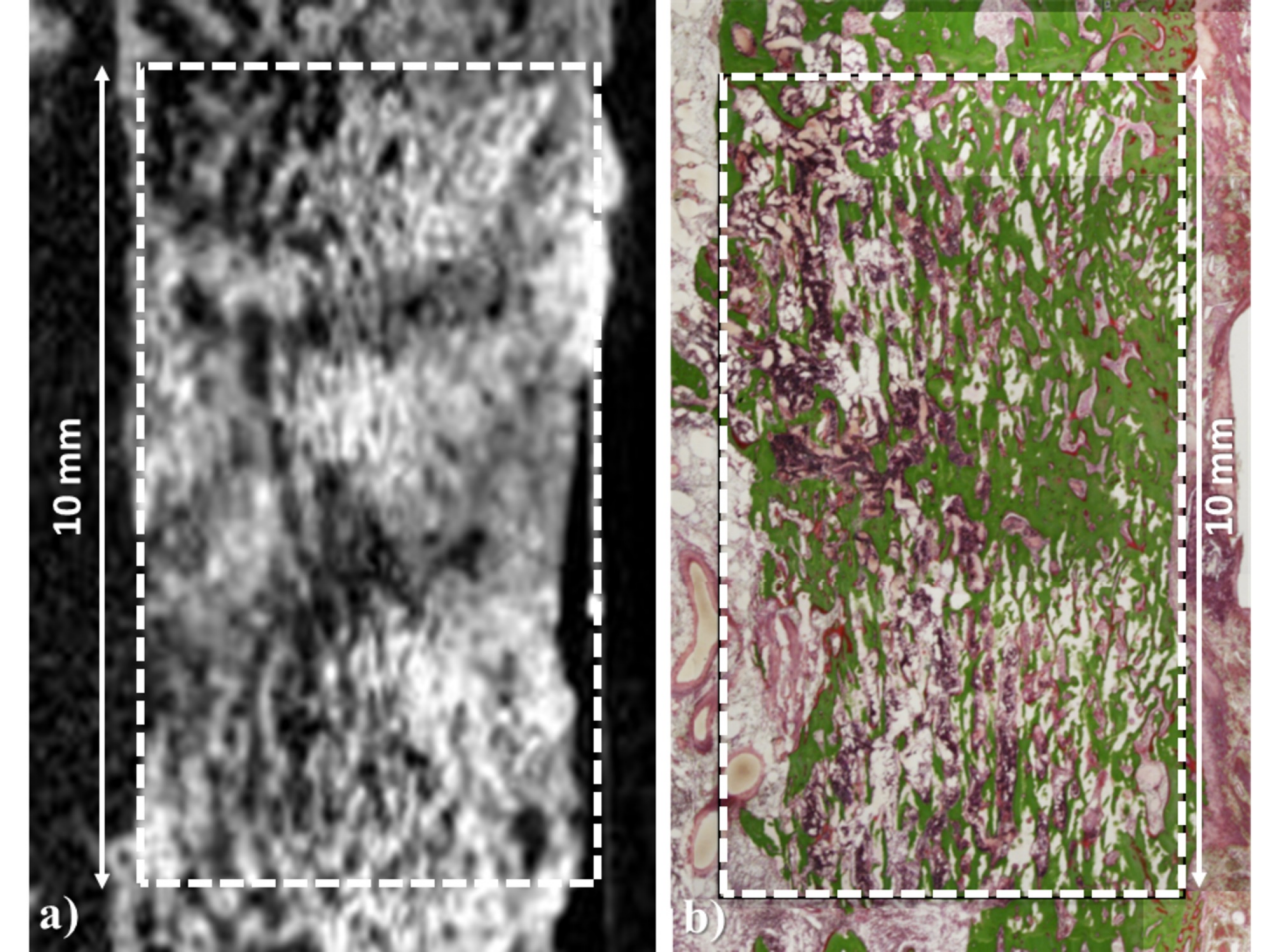
An implant experiment in a beagle model of a partial femur defect Ten-millimeter Affinos blocks were implanted into a partial cortical defect prepared in the femoral shaft in which the unidirectional porous direction was matched with the longitudinal axis of the femur. The bone defect was cross-bridged by new bone whose orientation matched the porous structure within three months. a: Micro CT; b: Villanueva Goldner staining Affinos: Kuraray Co., Ltd., Tokyo, Japan

The porosity of the material is 57% and the initial compressive strength is 8 MPa and 1.5 MPa in directions parallel and perpendicular to the pores, respectively [4]. The most distinctive feature of Affinos is that blood can reach the depths of the material rapidly by capillary action due to its unidirectional porous structure. It is also characterized by the presence of micropores, which are known to be beneficial for osteoconductivity, both on the surface and inside the material [4]. In tibial defect implant studies in rabbits, balanced resorption and regeneration with autologous bone were observed from an early stage of implantation; new bone and vascularization, as well as the presence of blood flow inside the material, were also observed [4-6]. Furthermore, in another femur defect implant study in a beagle, the porous structure of the material was filled by new bone within three months (Figure 2).

Affinos is also expected to present balanced absorption and regeneration similar to autologous bone from an early stage in clinical applications. However, limited clinical application studies have been reported so far [7-13]. Here, we report the benefits of Affinos as an absorbable material for bone regeneration by introducing clinical application cases in various fields of orthopedic surgery. We obtained consent to publish data from all the patients involved.

Case illustration 1 (spine)

**Figure 3:**
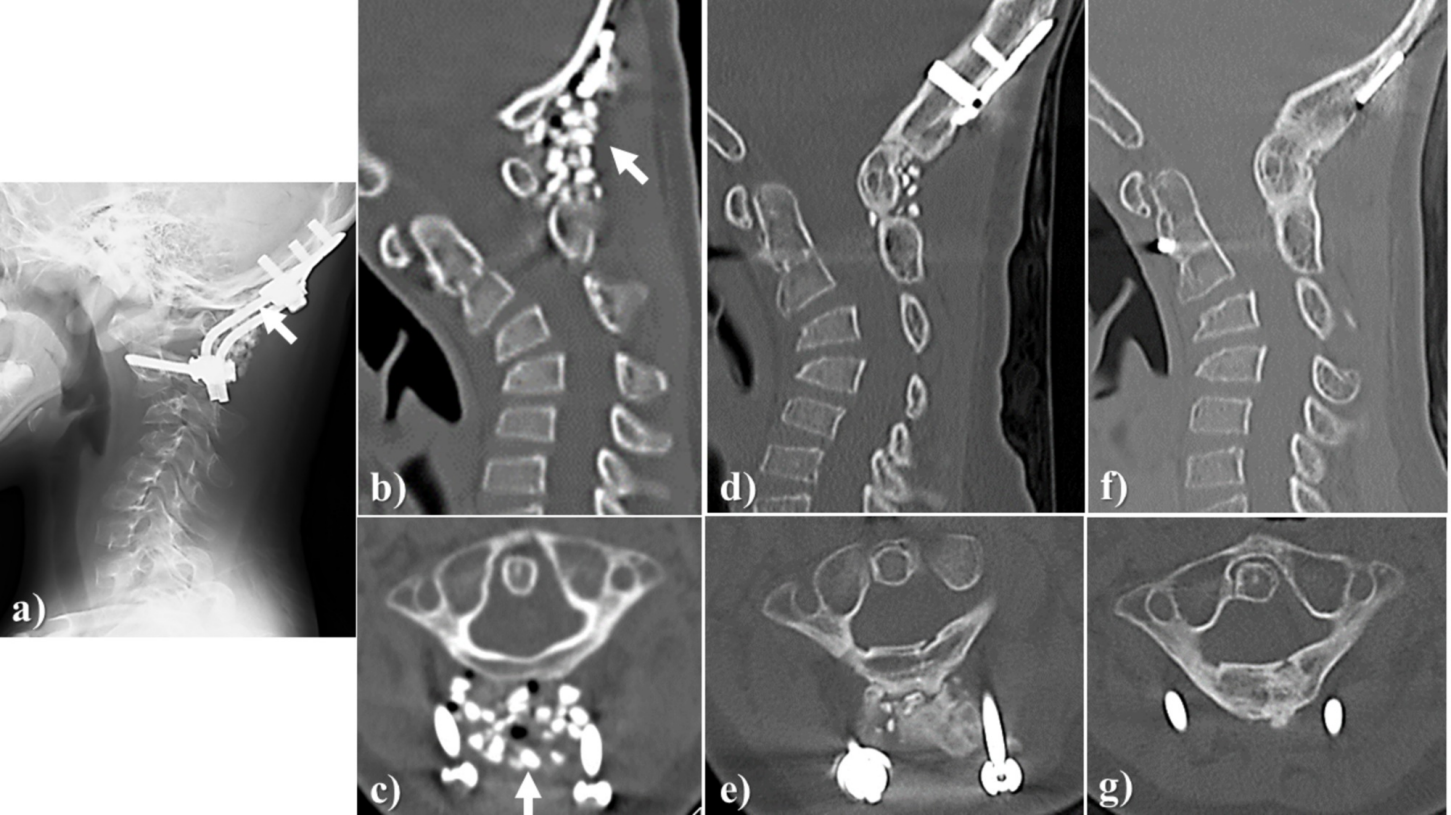
Case illustration 1 Posterior fusion was performed for a seven-year-old girl with chronic atlantoaxial rotatory fixation. a, b, c: The autologous ilium (the cancellous bone) and Affinos granules were mixed in approximately 1:1 ratio to fill the space between the occipital bone and the spinous process of the axis (arrow); d, e: At six months after surgery, favorable bone fusion and replacement with own bone were achieved; f, g: At 12 months after surgery, completion through sufficient remodeling was achieved. Affinos: Kuraray Co., Ltd., Tokyo, Japan

Posterior fusion was performed for a seven-year-old girl with chronic atlantoaxial rotatory fixation. In the procedure, the autologous iliac cancellous bone and Affinos granules were mixed in approximately 1:1 ratio to form a bone graft that filled the space between the occipital bone and the spinous process of the axis. At six months after surgery, favorable fusion and regeneration with natural bone were achieved and at 12 months post-surgery, completion through sufficient remodeling was achieved and the area from the occipital bone to the axis was completely fused (Figure 3).

Case illustration 2 (spine)

**Figure 4:**
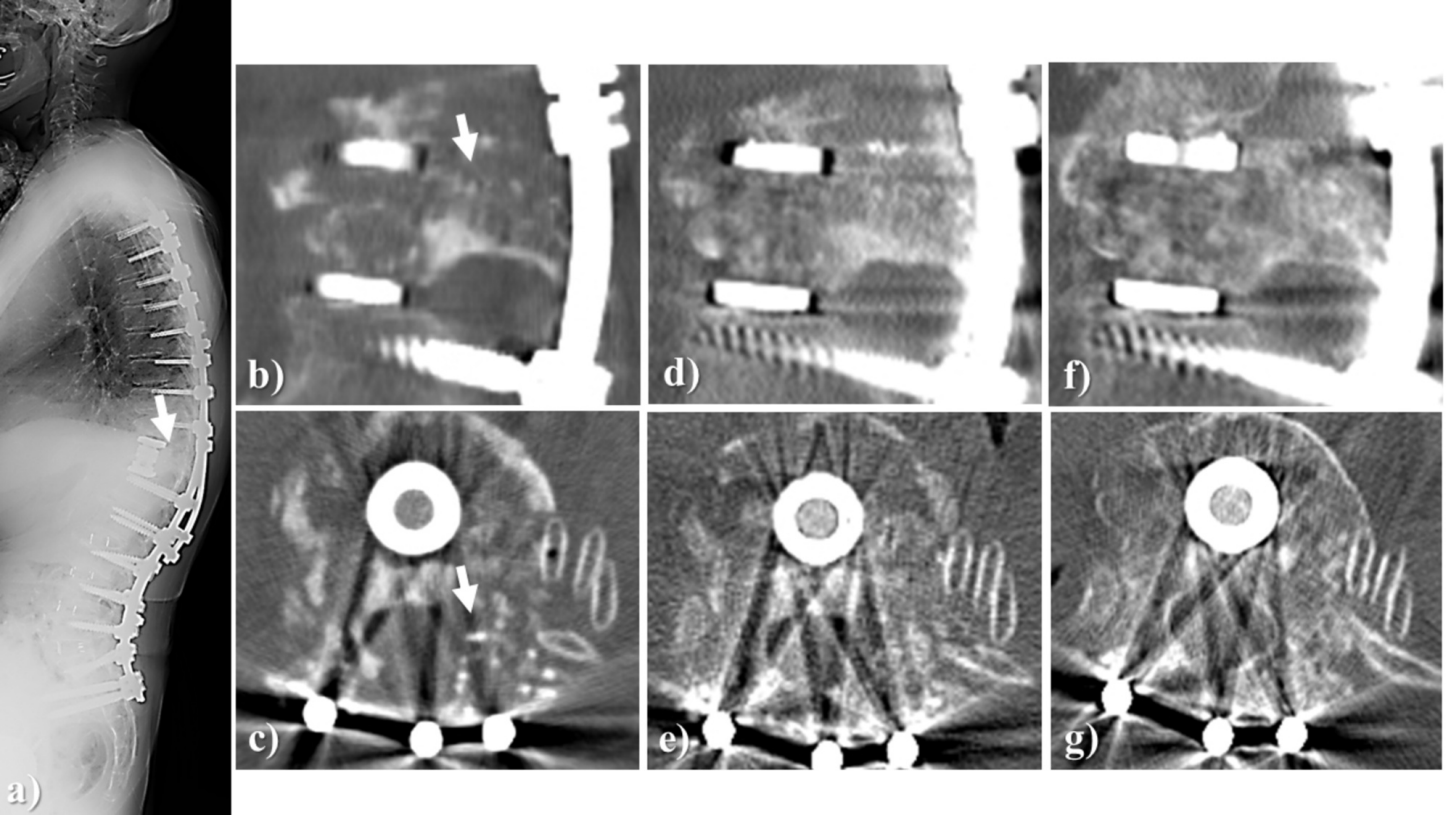
Case illustration 2 An anterior-posterior fusion was performed for a 74-year-old woman with advanced kyphotic deformity due to vertebral fracture at the upper end of the fusion and adjacent segmental disease after lumbosacral posterior fusion. a, b, c: X-ray of the whole spine and CT images of the left T12 pedicle immediately after surgery. The autologous iliac bone chips and Affinos granules were mixed in approximately 1:1 ratio to fill the bone defect generated by removing the pedicle of the fractured vertebra (arrow); d, e: At three months after surgery, artificial bone resorption and own bone formation progressed; f, g: At 12 months after surgery, the bone was regenerated as a new pedicle. Affinos: Kuraray Co., Ltd., Tokyo, Japan

Anterior-posterior fusion was performed for a 74-year-old woman with advanced kyphotic deformity due to vertebral fracture at the upper end of the fusion and adjacent segmental disease after lumbosacral posterior fusion. The autologous iliac bone chips and Affinos granules were mixed in approximately 1:1 ratio to form a bone graft that filled the bone defect generated by resecting the pedicle of the fractured vertebra on the extension of the posterior fusion for kyphosis correction. At three months after surgery, artificial bone resorption and new bone formation progressed. The bone was regenerated as a new pedicle 12 months after surgery (Figure 4).

Case illustration 3 (foot)

**Figure 5:**
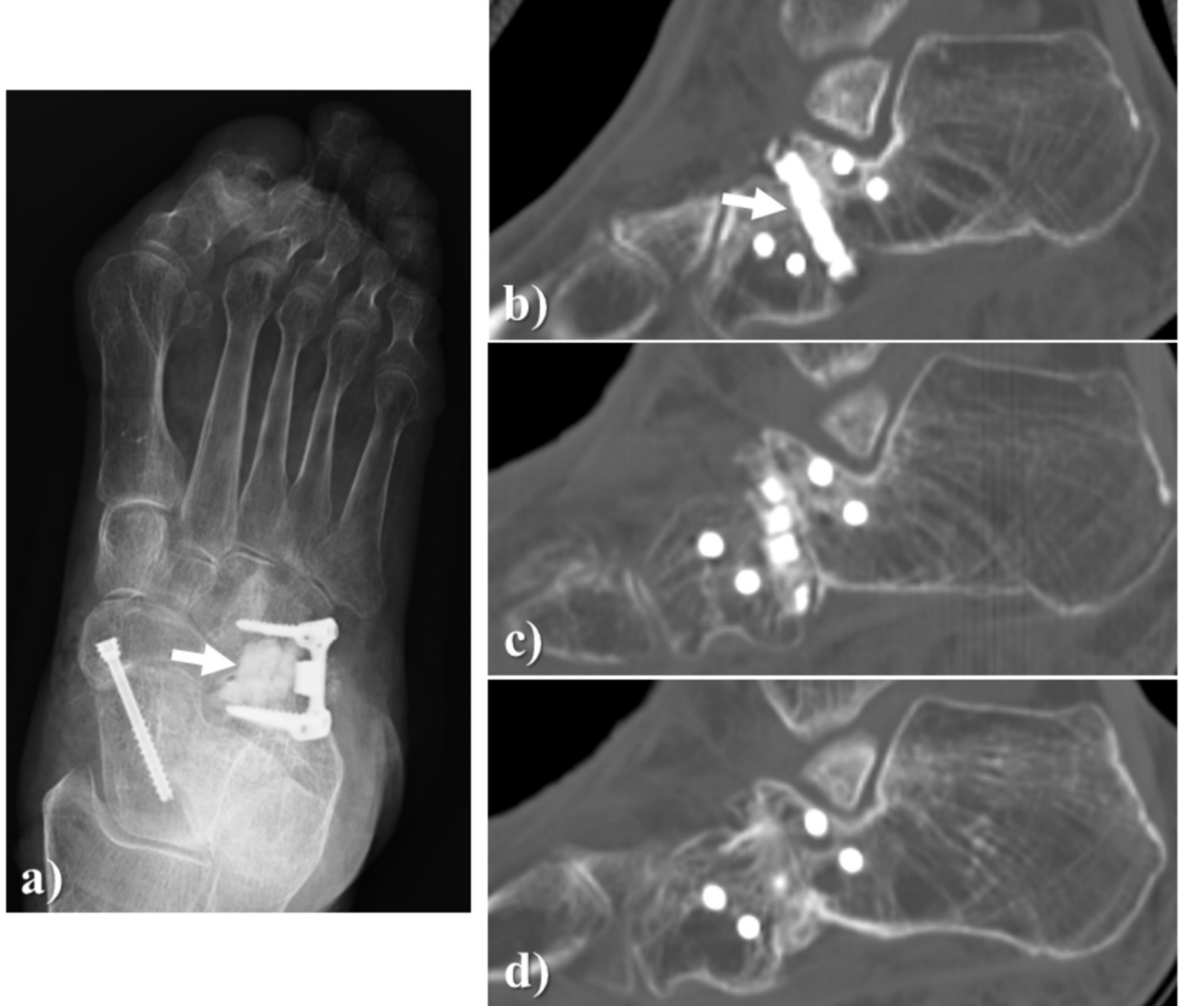
Case illustration 3 A 77-year-old woman with pes planovalgus deformity underwent a lateral column lengthening. a, b: The bony defect in the surgery was filled with Affinos blocks and granules (arrow); c: At three months after surgery, artificial bone resorption as well as own bone formation progressed; d: At 12 months after surgery, there was complete replacement with own bone. Affinos: Kuraray Co., Ltd., Tokyo, Japan

A 77-year-old woman with a pes planovalgus deformity underwent a lateral column-lengthening procedure. The bony defect in the surgery was filled with Affinos blocks and granules. At three months after surgery, artificial bone resorption, as well as new bone formation, progressed and at 12 months after surgery, there was complete regeneration with an autologous bone (Figure 5).

Case illustration 4 (benign bone tumor)

**Figure 6:**
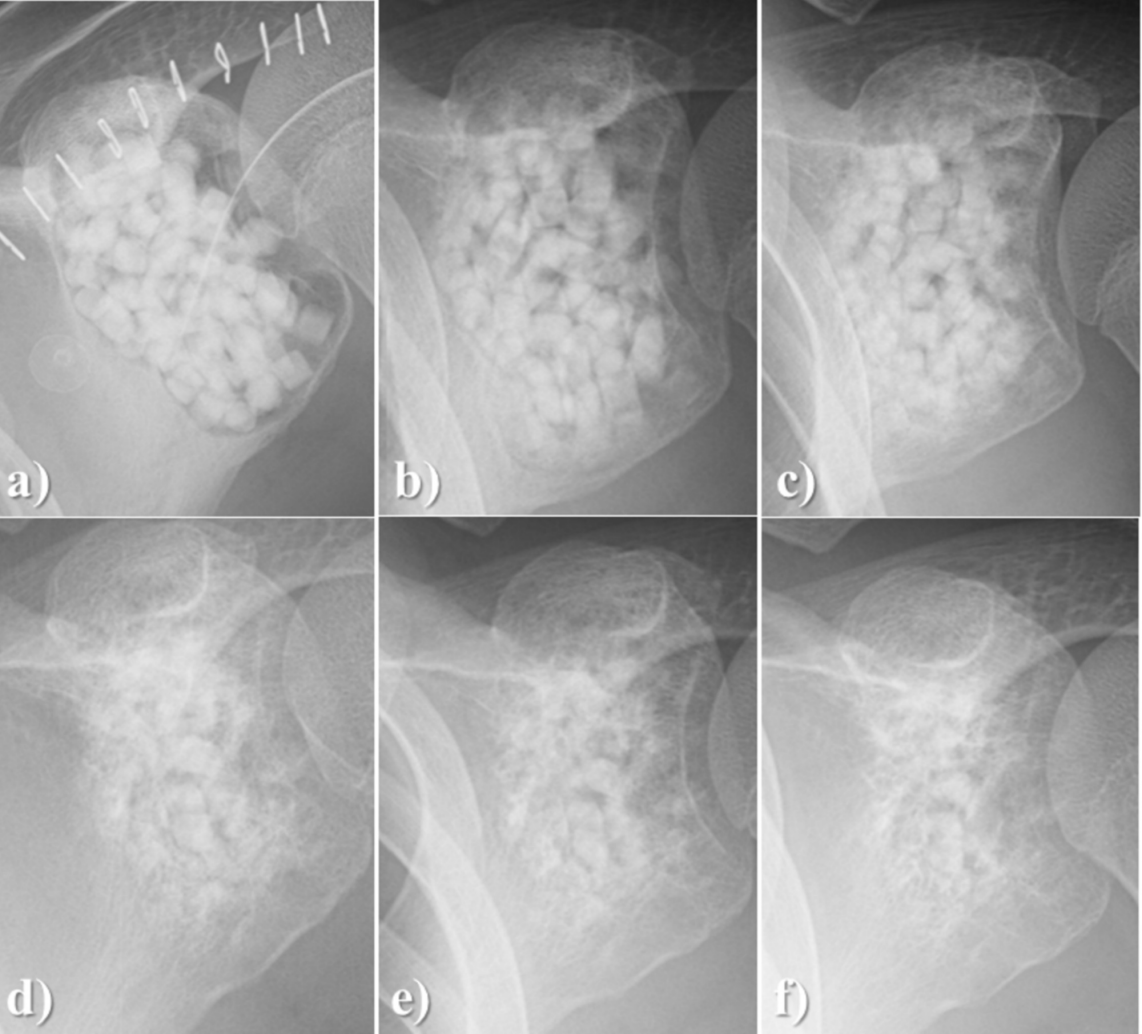
Case illustration 4 A 16-year-old man had a large bone defect originating from tumor curettage for chondroblastoma of the scapula. a: The bone defect was filled with Affinos granules (total weight, 20 g); b: At three months after surgery, artificial bone resorption from the marginal area was observed; c: At six months; d: At 12 months; e: at 18 months; f: at 24 months after surgery, the artificial bone graft was clearly replaced with the patient’s own bone Affinos: Kuraray Co., Ltd., Tokyo, Japan

A 16-year-old man had a large bone defect originating from tumor curettage for chondroblastoma of the scapula filled with Affinos. Despite the large number of granules being used (20 g), artificial bone resorption on the marginal area was observed 3 months after surgery. Moreover, it was observed that the bone graft was clearly replaced with the patient’s own bone over time (Figure 6).

Case illustration 5 (trauma)

**Figure 7:**
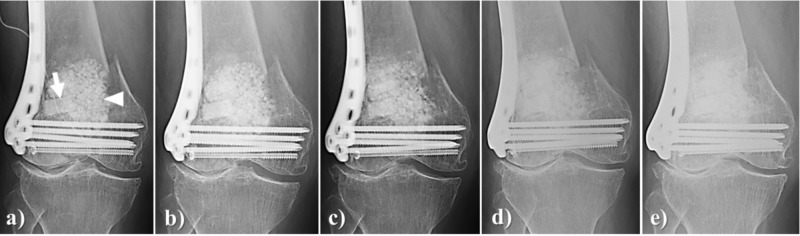
Case illustration 5 During surgery for supracondylar femur fracture, a large bone defect was observed in a 77-year-old woman. a: Large amounts of Affinos blocks (arrow) and granules (arrowhead) were used (total weight, approximately 25 g); b: At three months after surgery, resorption in the marginal area of the artificial bone block was observed; c: At six months after surgery, replacement with own bone in the marginal area was observed After bone fusion at the fracture site was completed, the state of gradual replacement with own bone over time was observed: d: 12 months; e: 24 months after surgery

During surgery for a supracondylar femur fracture, a large bone defect in a 77-year-old woman was filled by large amounts of Affinos blocks and granules (a total of approximately 25 g). At three months after surgery, resorption in the marginal area of the artificial bone block was observed and at 6six months after surgery, regeneration with autologous bone in the marginal area was observed. After boney fusion at the fracture site was completed, a state of gradual regeneration with natural bone over time (12 months and 24 months after surgery) was observed (Figure 7).

Case illustration 6 (bone graft donor site)

**Figure 8:**
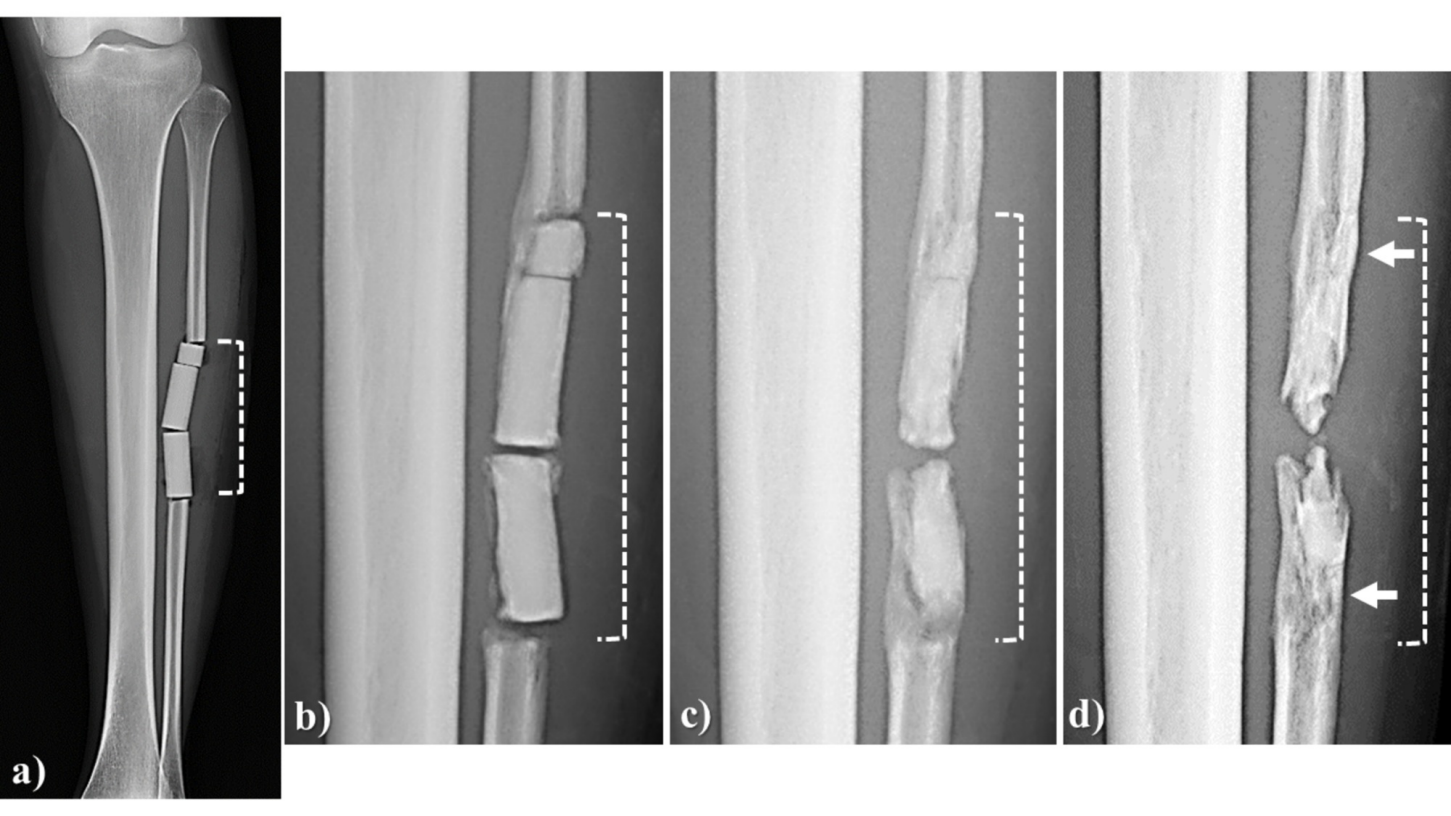
Case illustration 6 A 22-year-old woman had her fibula harvested and transplanted into the cervical spine for a 7.5 cm long defect (area indicated with a broken line). a: Affinos cylinder blocks were used in which the longitudinal and unidirectional pore directions were matched; b: At six months after surgery, fusion was achieved by new bone continuing from the stump of the fibula; c: At 12 months after surgery, fusion between blocks progressed and the area close to the stump was replaced with own bone; d: At 24 months after surgery, there was regeneration through the original tubular structure of the fibula in the area close to the stump (arrow). Affinos: Kuraray Co., Ltd., Tokyo, Japan

A 22-year-old woman had her fibula harvested and transplanted into the cervical spine for a 7.5 cm long defect. Affinos cylinder blocks with matched longitudinal and unidirectional pore directions were used. At six months after surgery, fusion was observed with new bone growing downward from the edge of the stump. At 24 months after surgery, the area proximal to the stump was completely regenerated with natural bone and some regeneration in the original tubular structure of the fibula was also seen (Figure 8).

Case illustration 7 (bone graft donor site)

**Figure 9:**
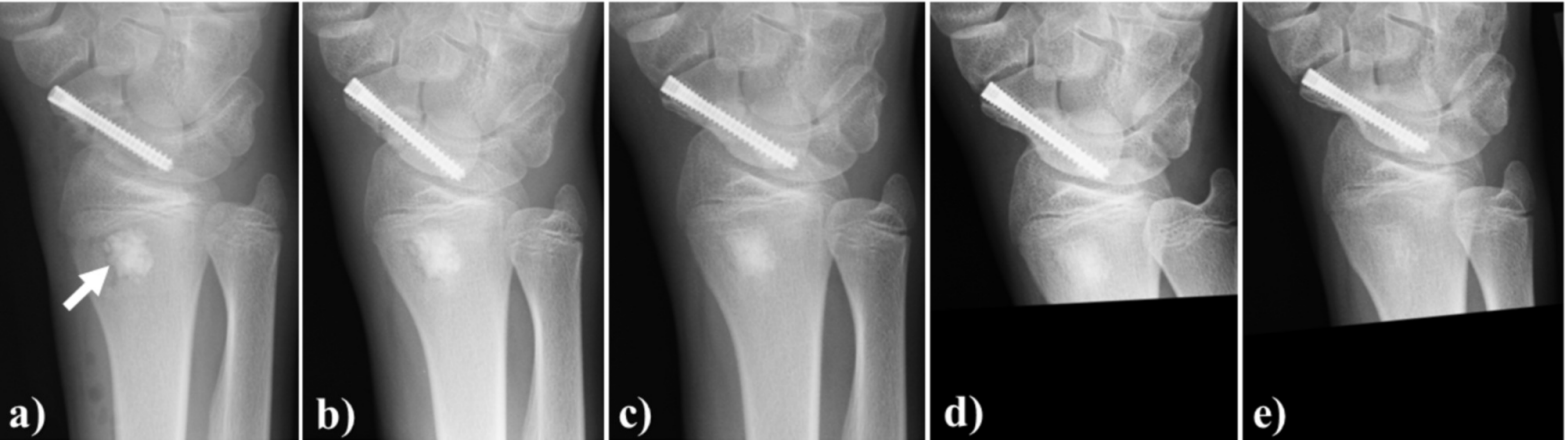
Case illustration 7 The cancellous bone of the distal radius in a 16-year-old man was transplanted to the nonunion after a scaphoid fracture. a: Affinos granules were used for the bone defect at the donor site (arrow); b: At one month after surgery, artificial bone resorption in the marginal area started; c: At three months after surgery, artificial bone resorption and replacement with own bone progressed; d: At six months after surgery, replacement with own bone progressed further; e: At 12 months after surgery, the bone graft was completely replaced with own bone and trabecular bone structure was regenerated Affinos: Kuraray Co., Ltd., Tokyo, Japan

The cancellous bone of the distal radius, in a 16-year-old man, was transplanted to the nonunion area at surgery after a scaphoid fracture. Affinos granules were used for the bone defect at the donor site. At three months after surgery, artificial bone resorption, as well as replacement with own bone, progressed and at 12 months after surgery, the bone graft was completely regenerated with natural bone (Figure 9).

## Discussion

Artificial bone is grafted to the cancellous bone area in the host bone in many conditions, such as in full bone defects, vertebral interbody fusions, tumor curettage areas, or nonunion areas, all of which have varying defect sizes and environments. The blood flow and local bone metabolism in the area to which an artificial bone is grafted depends on the diseased condition. For the clinical application cases of Affinos shown in this report, although the speeds of resorption and replacement with own bone varied, replacement with own bone and fusion were certainly achieved in all the cases and thus Affinos is an advantageous material for bone regeneration.

In order to further enhance the remarkable functions of Affinos as a resorbable artificial bone material, we have focused on its integration with teriparatide, bone marrow-derived cells, and platelet-rich plasma. We observed that new bone formation inside the material is promoted by teriparatide administration in an animal experiment [14]. Since Affinos allows bone marrow blood and platelet-rich plasma to rapidly penetrate material in depth by capillary action, these may contribute to the promotion of new bone formation inside the material [4,13]. Thus, Affinos is expected to become a novel absorbable biomaterial with beneficial features for bone regeneration in orthopedic surgery.

## Conclusions

Unidirectional porous β-TCP (Affinos) is a novel absorbable material for bone regeneration with a unique structure. It is useful in bone grafting in various orthopedic surgeries.
